# Exosomes from embryonic mesenchymal stem cells alleviate osteoarthritis through balancing synthesis and degradation of cartilage extracellular matrix

**DOI:** 10.1186/s13287-017-0632-0

**Published:** 2017-08-14

**Authors:** Yafei Wang, Dongsheng Yu, Zhiming Liu, Fang Zhou, Jun Dai, Bingbing Wu, Jing Zhou, Boon Chin Heng, Xiao Hui Zou, Hongwei Ouyang, Hua Liu

**Affiliations:** 1 Dr. Li Dak Sum & Yip Yio Chin Center for Stem Cell and Regenerative Medicine, Zhejiang University, Hangzhou, 310058 People’s Republic of China; 2 Key Laboratory of Tissue Engineering and Regenerative Medicine of Zhejiang Province, Hangzhou, 310058 People’s Republic of China; 3 Institute for Biomechanics, Swiss Federal Institute of Technology Zurich (ETH), Vladimir-Prelog-Weg 3, Zurich, 8093 Switzerland; 4 Endodontology, Faculty of Dentistry, The University of Hong Kong, Pokfulam, Hong Kong; 5 Central Laboratory, the First Affiliated Hospital, School of Medicine, Zhejiang University, Hangzhou, Zhejiang 310003 People’s Republic of China; 6 Department of Sports Medicine, School of Medicine, Zhejiang University, Hangzhou, 310058 People’s Republic of China; 7 State Key Laboratory for Diagnosis and Treatment of Infectious Diseases, Collaborative Innovation Center for Diagnosis and Treatment of Infectious Diseases, The First Affiliated Hospital, School of Medicine, Zhejiang University, Hangzhou, 310003 People’s Republic of China; 8 Harvard-MIT Division of Health Sciences and Technology, Brigham Women’s Hospital, Harvard Medical School, Boston, MA 02139 USA

**Keywords:** ESC, MSCs, Exosomes, Osteoarthritis

## Abstract

**Background:**

Mesenchymal stem cell therapy for osteoarthritis (OA) has been widely investigated, but the mechanisms are still unclear. Exosomes that serve as carriers of genetic information have been implicated in many diseases and are known to participate in many physiological processes. Here, we investigate the therapeutic potential of exosomes from human embryonic stem cell-induced mesenchymal stem cells (ESC-MSCs) in alleviating osteoarthritis (OA).

**Methods:**

Exosomes were harvested from conditioned culture media of ESC-MSCs by a sequential centrifugation process. Primary mouse chondrocytes treated with interleukin 1 beta (IL-1β) were used as an in vitro model to evaluate the effects of the conditioned medium with or without exosomes and titrated doses of isolated exosomes for 48 hours, prior to immunocytochemistry or western blot analysis. Destabilization of the medial meniscus (DMM) surgery was performed on the knee joints of C57BL/6 J mice as an OA model. This was followed by intra-articular injection of either ESC-MSCs or their exosomes. Cartilage destruction and matrix degradation were evaluated with histological staining and OARSI scores at the post-surgery 8 weeks.

**Results:**

We found that intra-articular injection of ESC-MSCs alleviated cartilage destruction and matrix degradation in the DMM model. Further in vitro studies illustrated that this effect was exerted through ESC-MSC-derived exosomes. These exosomes maintained the chondrocyte phenotype by increasing collagen type II synthesis and decreasing ADAMTS5 expression in the presence of IL-1β. Immunocytochemistry revealed colocalization of the exosomes and collagen type II-positive chondrocytes. Subsequent intra-articular injection of exosomes derived from ESC-MSCs successfully impeded cartilage destruction in the DMM model.

**Conclusions:**

The exosomes from ESC-MSCs exert a beneficial therapeutic effect on OA by balancing the synthesis and degradation of chondrocyte extracellular matrix (ECM), which in turn provides a new target for OA drug and drug-delivery system development.

**Electronic supplementary material:**

The online version of this article (doi:10.1186/s13287-017-0632-0) contains supplementary material, which is available to authorized users.

## Background

Osteoarthritis (OA) is a common joint disease worldwide, which exerts a significant detrimental effect on the patients’ life quality [1]. Pharmaceutical agents, particularly nonsteroidal anti-inflammatory drugs (NSAIDs) and paracetamol, have been widely applied in the clinic. However, their effects are largely limited to pain control with negligible effects on cartilage maintenance, which would ultimately lead to cartilage destruction [2, 3]. Although surgical interventions such as total knee or hip arthroplasty could partially overcome pain and deformity in end-stage OA patients, this could also cause further complications such as increased risks of infection, thrombus formation and secondary surgery, particularly in elderly patients [4–6]. Hence, there is a dire need to develop new strategies to protect cartilage and attenuate OA development, particularly minimally invasive interventions at the early stages of OA.

Cell transplantation is an emerging therapeutic modality for OA treatment, and mainly involves the utilization of autologous chondrocytes and mesenchymal stem cells [7, 8]. Although autologous chondrocytes as the main cell type in cartilage may provide a safe and efficacious solution, these have the inherent drawbacks of limited availability, de-differentiation and function loss during the process of in vitro expansion [8]. Therefore, the application of mesenchymal stem cells (MSCs) for OA therapy has attracted much more attention from scientific investigators [7, 9–11]. Clinical applications of adult MSCs are limited by donor source and individual physical conditions [12–14]. MSCs derived from pluripotent embryonic stem cells (ESC-MSCs) have become another potential and promising source of MSCs. The unlimited self-renewal capacity and pluripotency of ESCs [15] in turn ensures less inter-batch variability and a more consistent supply of ESC-MSCs. Furthermore, as a cell source for the treatment of various diseases, ESC-MSCs have also been reported to possess immunomodulatory properties, as well as regenerative potential, similar to adult MSCs [16]. However, there have not yet been any studies that have investigated their therapeutic effects on OA and probed the underlying mechanisms involved.

Besides the well-documented immunomodulatory properties and multidifferentiation capacity of MSCs [16–18], the communication and interaction between MSCs and chondrocytes, either by direct cell-cell contact or indirectly via secretions such as exosomes, have aroused much interest [11, 19–22].

As one of the major pathways of extracellular signaling, exosomes have been implicated in many diseases and participate in many physiological processes. These are derived from fusion in cell to cell communication, as well as serve as carriers of genetic information [23]. Recently, exosomes derived from MSCs have received much attention due to a study showing that severe graft versus host disease (GVHD) can be treated with MSCs-derived extracellular vesicles [24], thus suggesting that the observed therapeutic effects of MSCs can in fact be reproduced by MSCs-derived exosome treatment [19, 25].

In this study, we report the positive therapeutic effects of ESC-MSCs on osteoarthritis, and investigate the underlying mechanisms involved.

## Methods

### Mice and OA model

Thirty-two 2-month-old C57BL/6 J mice purchased from the animal center of Zhejiang University were utilized for all experiments. The mice were anesthetized with sodium pentobarbital (8 mg/ml, 1 ml/100 g) and randomly distributed into experimental and control groups. Among them, 12 mice were in the sham group, and 20 mice were utilized for the experimental OA model, which was induced by destabilization of the medial meniscus (DMM) surgery, following the transection of the medial meniscotibial ligament as previously described [26]. In the first animal section, DMM surgery was performed in the bilateral knee joints and five mice were allocated to each group. Three mice were allocated to each sham group. OA pathology developed gradually throughout 4 weeks before single intra-articular injection of 5 μL ESC-MSCs (1 × 10^6^/joint) or 5 μL PBS per joint with a microliter syringe (Hamilton Company, Reno, NV, USA, 1702) and 5 mm 30-gauge needles (Hamilton Company, 7803-05). In the second animal section, DMM surgery was performed in the bilateral knee joints and five mice were allocated to each group. Three mice were allocated to each sham group. OA pathology developed gradually throughout 4 weeks before multiple injections of 5 μL exosomes isolated from ESC-MSCs per joint or 5 μL PBS per joint, which was performed every 3 days for 4 weeks with a microliter syringe (Hamilton Company, 1702) and 5 mm 30-gauge needles (Hamilton Company, 7803-05).

### Cell culture

MSCs were derived from the male H1 human ES cell line (ESC) that was obtained from WiCell Corporation. Madison, WI, USA (http://www.wicell.org), which was maintained in the undifferentiated state by culture in a feeder-free system. ESC-MSCs were obtained as described in our previous study [27]. Briefly, ESCs were detached from Matrigel (BD, Franklin Lakes, NJ, USA, Cat. #354234) coated plates by a 10-minute (min) incubation with 0.02% (w/v) EDTA (Sigma-Aldrich, St. Louis, MO, USA, Cat. #E8008) and seeded onto a gelatinized 10-cm plates. The cells were cultured in L-DMEM (Life Technologies, Carlsbad, CA, USA, Cat. #11885084) supplemented with 10% (v/v) fetal bovine serum (FBS; Life Technologies, Cat. #10099-141). The cells were then trypsinized (0.05% (w/v), Life Technologies, Cat. #15400-054) upon reaching 80% confluency and then plated at a density of 10^3^ cells/cm^2^. The medium was pre-processed to delete the suspension in FBS with centrifugation at 110,000 × g overnight at 4 °C, according to the published protocol of Thery et al. [28]. Cells between passage number 4 and 7 were utilized.

### Chondrocyte isolation and culture

Primary chondrocytes were obtained from nine C57BL/6 mice within 2 days after birth. The articular cartilage derived from the terminal of tibia and femur was digested with 0.2% (w/v) type II collagenase [29, 30], then expanded in medium containing F12 (Life Technologies, Cat. #88215) with 10% (w/v) FBS (Life Technologies, Cat. #10099-141), and 1% (w/v) penicillin/streptomycin (Life Technologies, Cat. #15140-122). Chondrocytes at the first passage were seeded into 24-well plates containing glass slides for immunofluorescence analysis and six-well plates for western blot analysis. At 24 hours after cell seeding, we changed the medium into that with or without 2 ng/mL interleukin 1 beta (IL-1β) (PeproTech, Rocky Hill, NJ, USA) according to the experimental design. Forty-eight hours later, the cells were collected for analysis. Cells within 3 passages were utilized.

### Colony-forming unit assay

ESC-MSCs were seeded at approximately 20 cells/cm^2^ in triplicates and cultured for up to 21 days with regular medium replenishment every 3–4 days. The colonies were stained with 1% (w/v) crystal violet (Sigma-Aldrich, Cat. #3886) in methanol for 10 min.

### Flow cytometry

Cells were harvested by trypsinization (0.05% (w/v); Life Technologies, Cat. #15400-054) for 2 min, and the cell pellet was resuspended in PBS to a titer of 10^6^/100 μL. The cell suspension was incubated with the following antibodies respectively. CD34-FITC (Miltenyi Biotec, Bergisch Gladbach, Germany, Cat. #130-098-142), CD45-PE (Miltenyi Biotec, Cat. #130-080-201), CD73-PE (Miltenyi Biotec, Cat. #130-112-060), CD90-FITC (Miltenyi Biotec, Cat. #130-095-403) and CD105-FITC (Miltenyi Biotec, Cat. #130-112-327). About 2 μL of each antibody (1:10 diluted according to the datasheet of the antibodies) per tube was added and incubated for 10 min in darkness at 2–8 °C. Cells were washed with PBS completely and resuspended in 1% (w/v) paraformaldehyde. Samples were run on a FC500MPL flow cytometer (Beckman Coulter, Brea, CA, USA) and the data were analyzed by FlowJo vX.0.7 software (FlowJo LLC, Ashland, OR, USA).

### Trilineage differentiation assay

We tested the multidifferentiation potential of the ESC-MSCs toward the osteogenic, adipogenic, and chondrogenic lineages in vitro as previously described [31, 32]. Briefly, osteogenic differentiation of MSCs was induced with 10% (v/v) FBS, 1 mM β-glycerol phosphate, 10^-8^ M dexamethasone, and 50 μg/mL ascorbic acid. Adipogenic differentiation was induced with 10% (v/v) FBS, 500 μM 1-methyl-3-isobutylxanthine, 10^-9^ M dexamethasone, and 60 μM indomethacin. Chondrogenic differentiation was induced in micromass culture in the presence of 10 ng/mL TGF-β3 (PeproTech), 50 μg/mL ascorbic acid, 1% (v/v) insulin, transferrin, and selenium solution (Life Technologies, Cat. #41400045), 10^-7^ M dexamethasone, 100 μM sodium pyruvate (Gibco, Cat. #11360070). Induction to these lineages was stopped after 3 weeks and then assessed by Alizarin red staining, oil red staining, and Safranin O staining respectively.

### Isolation of exosomes from ESC-MSCs medium

The exosomes were isolated from 600 mL of ESC-MSCs culture medium, which were collected when cells have attained around 60% confluency, and were finally re-suspended in 600 μL PBS. The isolation and purification followed the multistep ultracentrifugation process as previously described [28]. Briefly, the conditioned medium containing exosomes (CM + Exo) was obtained by centrifugation at 2000 × g for 10 min at 4 °C to remove dead cells and 10,000 × g for 30 min at 4 °C to remove cell debris. This was followed by centrifugation at 110,000 × g for 90 min at 4 °C to separate the exosomes from the CM + Exo. At the same time, the CM without exosomes (CM-Exo) was also collected. The exosomes were washed with PBS and subjected to a final centrifugation, prior to being stored in PBS at -80 °C.

For tracking, 100 μL of exosomes were transferred to a 1.5 mL tube, and were incubated with 10 μM DiI (Beyotime, Shanghai, China, Cat. #C1036) at 37 °C for 15 min, washed with PBS at 110,000 × g for 90 min at 4 °C, and then stored in 100 μL PBS at -80 °C.

### Western blot assay

Cellular protein was extracted with RIPA lysis buffer (Solarbio, Beijing, China, Cat. #R0010), and the total protein concentration was determined with a BCA Protein Assay Kit (Pierce, Rockford, IL, USA, Cat. #23225). The 20 μg extracted cellular protein was loaded on 10% (w/v) SDS-PAGE-denaturing gels. After electrophoresis, the proteins were transferred to a polyvinylidene difluoride membrane and blocked in 5% (w/v) bovine serum albumin (BSA, Sangon Biotech, Shanghai, China, Cat. #9048-4b-8) for 1 h at room temperature. The membrane was incubated overnight at 4 °C with mouse anti-collagen type II (Col II) (1:500; COL2A1, Santa Cruz Biotechnology, Dallas, TX, USA, Cat. #sc-52658), rabbit anti-ADAMTS5 (1:250; Abcam, Cambridge, MA, USA, Cat. #ab41037) or mouse anti-GAPDH (1:1000; Beyotime, Cat. #AG019) antibody. After washing in Tris-buffered saline with Tween-20 (TBST), the horseradish peroxidase (HRP) secondary antibodies: HRP-labeled goat anti-mouse IgG (1:1000; Beyotime, Cat. #A0216) and goat anti-rabbit IgG antibody, peroxidase-conjugated (1:1000; EMD Millipore, Billerica, MA, USA, Cat. #AP132P) was diluted in 5% (w/v) BSA solution and incubated accordingly with the membrane for 1 h at room temperature (RT). The excessive secondary antibody was washed off by TBST, and a chemiluminescent signal was generated by the ECL Imaging Kit (Thermo Fisher Scientific, Waltham, MA, USA, Cat. #32209).

Exosomes (1:1000) and supernatant of ESC-MSCs were suspended in sample loading buffer solution (without 2-mercaptoethanol). Protein concentration was determined by Micro BCA™ Protein Assay Kit (Pierce, Cat. #23235). Equal volume of protein samples (40 μL) were loaded on 12% (w/v) SDS-PAGE-denaturing gels. After electrophoresis, protein was transferred to a polyvinylidene difluoride membrane and blocked in 5% bovine serum albumin (BSA, Sangon Biotech, Cat. #9048-4b-8) for 1 h at RT. The membrane was incubated overnight at 4 °C with rabbit anti-CD9 (1:2000; Abcam, Cat. #ab92726) or mouse anti-CD63 (1:200; Santa Cruz Biotechnology, Cat. #sc-31211) antibody. After washing in Tris-buffered saline with Tween-20(TBST), the horseradish peroxidase (HRP) secondary antibodies such as peroxidase-conjugated goat anti-rabbit IgG antibody (1:1000; EMD Millipore, Cat. #AP132P) and HRP-conjugated donkey anti-goat IgG antibody (1:1000; R&D Systems, Minneapolis, MI, USA, Cat. #HAF109) were diluted in 5% BSA solution and incubated accordingly with the membrane for 1 h at RT. The excessive secondary antibody was washed off by TBST, and a chemiluminescent signal was generated by ECL imaging kit (FDbio-Femto ECL, Belfort, France, Cat. #FD8030).

### Immunocytochemistry

The chondrocytes were fixed in 4% (w/v) buffered paraformaldehyde solution for 15 min, followed by permeabilization with 0.2% (w/v) Triton X-100 for 10 min. The fixed cells were then incubated with primary antibodies: mouse anti-Col II antibody (1:100; EMD Millipore, Cat. #MAB8887) and rabbit anti-ADAMTS5 (1:100; Abcam, Cat. #ab41037) at 4 °C overnight and were washed with PBS thoroughly. Subsequently, these cells were incubated with the corresponding goat anti-mouse IgG (H + L) cross-adsorbed secondary antibody, Alexa Fluor 488 (1:250; Invitrogen, Carlsbad, CA, USA, Cat. #A11001) and F(ab’)2-goat anti-rabbit IgG(H + L) cross-adsorbed secondary antibody, Alexa Fluor 555 (1:250; Invitrogen; Cat. #21430), respectively. Finally, the cell nuclei were counterstained by DAPI (1:5000; Beyotime; Cat. #C1002) and imaged under confocal microscopy (Olympus, Tokyo, Japan, BX61W1-FV1000). The DAPI-positive cells were counted as the total cell number (X), while the cells stained with green fluorescence were counted as the Col II-positive cell number (Y). The Col II-positive cell percentage = Y/X*100%. Three independent staining tests were performed. Five visual fields were randomly selected from each sample and the cells were counted by a technician in a single blinded manner. The immunofluorescence intensity of ADAMTS5 was quantified by the ImageJ software (ImageJ v2.1.4.7, National Institutes of Health, Bethesda, MD, USA).

### Tissue histology and immunohistochemistry

Mouse joints were isolated and fixed in 4% buffered paraformaldehyde for 24 h, then decalcified in 10% (w/v) EDTA (pH 7.4) for 21 days (d) at 4 °C before being embedded in paraffin. Sagittal joint sections at 6 μm thickness were processed for Safranin O and Fast Green staining. Cartilage destruction was evaluated using the Osteoarthritis Research Society International (OARSI) scoring system according to the percentage of the vertical clefts/erosion to the calcified cartilage [26]. All four parts of the joint: medial femoral condyle, lateral femoral condyle, medial tibial plateau and lateral tibial plateau presented in the figures were semi-quantitatively scored according to the recommended OA grading table (Table 1) supplied by Glasson et al*.* The OA grading of each joint is expressed as the maximum or summed score of the four quadrants, respectively. Immunohistochemical staining was performed using a standard protocol. After dewaxing, heat-induced antigen retrieval was performed in retrieval solution overnight at 64 °C. The solution was composed of 0.1 M trisodium citrate (20.5 mL) and 0.1 M citric acid anhydrous (4.5 mL) in 225 mL distilled water. Sections were incubated overnight at 4 °C with primary antibodies: rabbit anti-ADAMTS5 (1:100; Abcam; Cat. #ab41037), mouse anti-Col II (1:50; COL2A1, Santa Cruz Biotechnology, Cat. #sc-52658), rabbit anti-aggrecan neoepitope antibody (1:100; Novus Biologicals, Littleton, CO, USA, Cat. #NB100-74350SS). After washing off excess primary antibodies, these samples were incubated with secondary antibodies conjugated with HRP: HRP-labeled goat anti-mouse IgG (1:200; Beyotime, Cat. #A0216) and goat anti-rabbit IgG antibody, peroxidase-conjugated (1:600; EMD Millipore, Cat. #AP132P) was diluted in 1% (w/v) BSA solution and incubated the section for 1 h at room temperature (RT). DAB detection system (Solarbio, Cat. #DA1010) were used to visualized the section. The stained specimens were photographed digitally under a slide scanning machine (Pannoramic MIDI, 3DHISTECH Ltd., Budapest, Hungary).

**Table 1:** The OA Grading Table

Scores	Osteoarthritis damage
0	Normal
0.5	Loss of Safranin O without structural changes
1	Small fibrillations without loss of cartilage
2	Vertical clefts down to the layer immediately below the superficial layer and some loss of surface lamina
3	Vertical clefts/erosion to the calcified cartilage extending to <25% of the articular surface
4	Vertical clefts/erosion to the calcified cartilage extending to 25–50% of the articular surface
5	Vertical clefts/erosion to the calcified cartilage extending to 50–75% of the articular surface
6	Vertical clefts/erosion to the calcified cartilage extending >75% of the articular surface

### Transmission electron microscopy

Purified exosomes were fixed with 1% (w/v) glutaraldehyde in PBS (pH 7.4). After rinsing, a 20 μL drop of the suspension was loaded onto a formvar/carbon-coated grid, negatively stained with 3% (w/v) aqueous phosphotungstic acid for 1 min, and then imaged under transmission electron microscopy (HT7700, Hitachi, Tokyo, Japan). The diameter of the exosome was evaluated by the ImageJ software (ImageJ v2.1.4.7, National Institutes of Health), and we analyzed the diameter distribution and percentage values with the Excel software (Microsoft (Redmond, WA, USA) Excel for Mac version15.24).

### Quantitative real-time polymerase chain reaction (qRT-PCR)

Total RNA was isolated from mouse primary chondrocytes by lysis in TRIzol (Takara, Shigo, Japan, Cat. #9109). The reverse transcription process applied ReverTra Ace qPCR RT Master Mix (Toyobo, Osaka, Japan, Cat. #FSQ-201). The qRT-PCR was performed utilizing Brilliant SYBR Green QPCR Master Mix (Takara, Cat. # RR420A) with a LightCycler apparatus (480II, Roche, Mannheim, Germany). The amplification efficiencies of primer pairs were validated to enable quantitative comparison of gene expression. All primer sequences (Invitrogen) were designed using Primer 5.0 software (see Additional file 1). Each qRT-PCR was performed three times on at least three different experimental replicates, and results were normalized to those obtained with the endogenous reference gene (Gapdh).

### Statistical analysis

All data were expressed as mean ± SE unless otherwise stated. All experiments in vitro were repeated independently at least twice in addition to the triplicates applied in each experiment. Statistical results were analyzed and bar charts were constructed with GraphPad Prism version 5.0 (GraphPad Software, San Diego, CA, USA). Statistical results were considered significant when the *p* value was less than 0.05. Two-tailed Student’s *t* test was used to compare two groups at the same time point. One-way ANOVA including the Tukey-Kramer post hoc test was used to compare multiple groups at the same time point. Experimental data of the in vivo experiment was analyzed by the Mann-Whitney test with the SPSS software (IBM Corp., Armonk, NY, USA).

## Results

### Establishment of ESC-MSCs

**Fig. 1:**
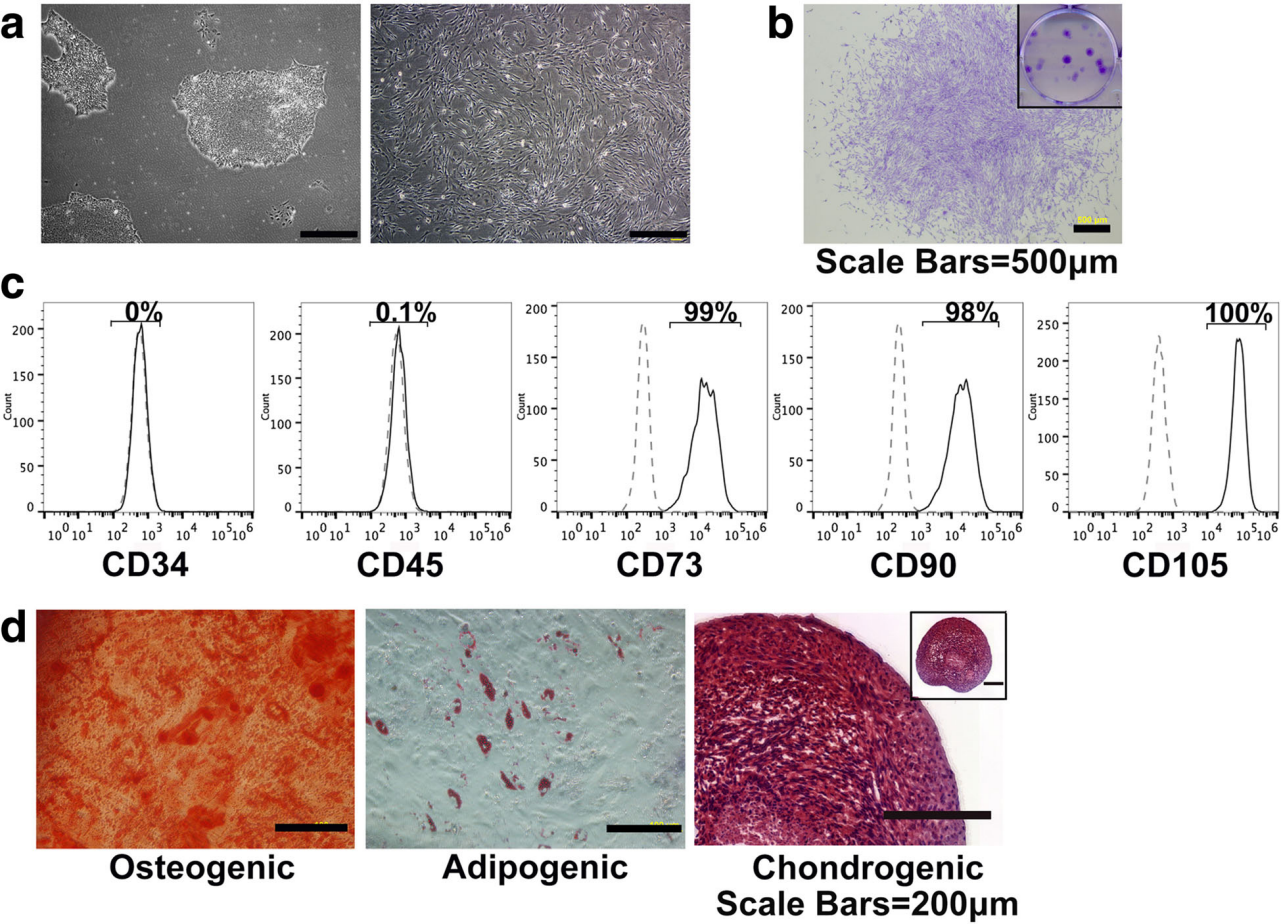
Establishment and identification of ESC-MSCs. **a** Morphology of the ESCs (*left*) and induced MSCs (*right*). Scale bars, 500 μm. **b** Colony formation by ESC-MSCs. Gross view of the whole well was shown in the small image. Scale bars, 500 μm. **c** Surface molecular profile of the ESC-MSCs, which were negative in CD34 and CD45 expression, but positive in CD73, CD90, and CD105 expression. The *dotted lines* indicate isotype-matched mouse IgG Ab control staining. **d** Identification of osteogenic, chondrogenic and adipogenic cells by Alizarin red staining, oil red staining and Safranin O staining respectively. Scale bars, 200 μm. *ESC-MSCs* embryonic stem cell-derived mesenchymal stem cells

The human ESC is a cell line obtained from WiCell Corporation. The ESCs cultured on Matrigel yielded compact colonies with sharp borders (Fig. 1a). These cells, however, adopted a fibroblast-like morphology upon plastic adhesion. Compared with ESCs, these cells displayed spindle-shaped morphology in monolayer culture and were distributed sparsely (Fig. 1a). When these cells were seeded at densities as low as 200 cells per 9.5 cm^2^ well, they developed into round and tight colonies (Fig. 1b). Flow cytometry analysis showed that more than 95% of these cells expressed the classical MSC markers including CD73, CD90, and CD105, while no cells expressed hematopoietic markers such as CD34 and CD45 (Fig. 1c). Upon osteogenic inducement, these cells synthesized matrix which were stained dark brown by Alizarin red, indicating calcium deposition. After adipogenic induction, these cells displayed round orange droplets within the cytoplasm upon oil red staining, which indicates formation of oil droplets. The cells were kept in aggregates before chondrogenic differentiation, resulting in formation of a pellet and synthesized matrix which were stained orange and red by Safranin O staining, thus indicating cartilage matrix formation (Fig. 1d).

### Alleviation of osteoarthritis by ESC-MSCs

**Fig. 2:**
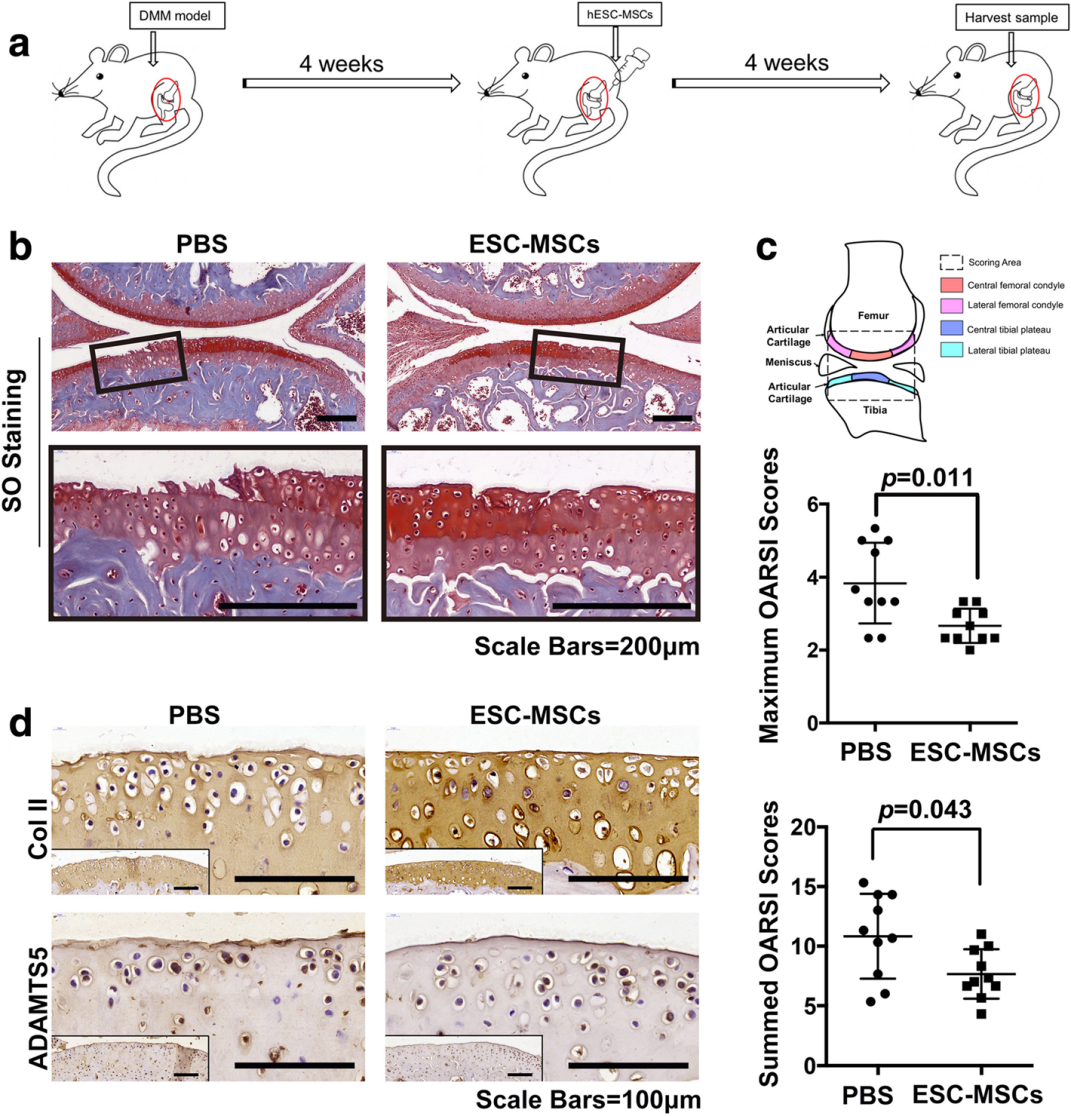
Recovery of cartilage destruction by intra-articular injection of ESC-MSCs in a DMM model. **a** Flowchart of the in vivo experiment. **b** The Safranin O and Fast Green staining of cartilage destruction at 8 weeks after surgery. *Boxed regions* were magnified and shown in the next line. Scale bars, 200 μm. **c** OARSI scores of cartilage destruction. n = 10 in each group. **d** Immunohistochemical staining of COL II and ADAMTS5. *Boxed regions* show the gross view. Scale bars, 100 μm. *COL II* collagen type II, *ESC-MSCs* embryonic stem cell-derived mesenchymal stem cells, *OARSI* Osteoarthritis Research Society International

To evaluate the therapeutic effects of ESC-MSCs on osteoarthritis, we injected 5 μL of cell suspension (1 × 10^6^) directly in the bilateral joints of five OA mice at 4 weeks after DMM surgery as the ESC-MSC group and injected 5 μL of PBS in the bilateral joints of five OA mice at 4 weeks after DMM surgery as the control group. We harvested the joint samples after the following 4 weeks (Fig. 2a). Histological evaluation revealed that the PBS-injected DMM mice have more sever fibrillation and erosion of the calcified cartilage. The maximal OARSI score was 3.8 ± 1.04, and the summed OARSI score was 10.8 ± 3.37. However, the ESC-MSC-injected DMM mice exhibited less fibrillation with vertical clefts only down to the layer immediately below the superficial layer, and some loss of surface lamina. The maximal OARSI score was 2.6 ± 0.45, and the summed OARSI score was 7.6 ± 1.97 (Fig. 2b). Hence, the OARSI evaluation in the ESC-MSCs group revealed significantly lower maximal and summed scores than the control group (*p* = 0.011 and 0.044 respectively) (Fig. 2c) (see Additional files 2 and 3). The immunohistochemistry results showed that the cartilage of the ESC-MSCs group exhibited much stronger Col II-specific staining, much weaker ADAMTS5-specific staining and aggrecan neoepitope-specific staining than the control group (Fig. 2d) (see Additional file 2).

### Effect of ESC-MSCs culture medium deficient in exosomes on cartilage matrix maintenance

**Fig. 3:**
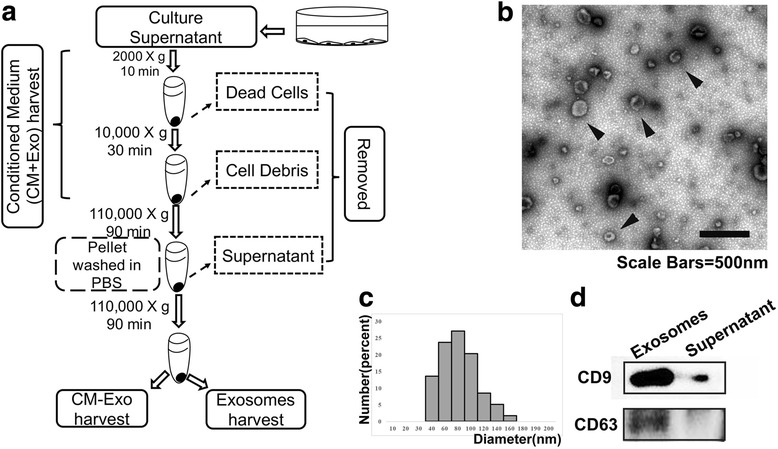
Isolation and identification of exosomes. **a** Flowchart of conditioned medium (CM + Exo), exosomes and conditioned medium without exosomes (CM-Exo) preparation. **b** Exosomes morphology. Scale bar, 500 nm. **c** The diameter distribution of isolated exosomes. **d** Expression of CD9 and CD63 by exosomes and supernatant

To investigate whether exosomes or cytokines exert a critical effect on the alleviation of OA by ESC-MSCs, we harvested the conditioned medium (CM + Exo), the conditioned medium without exosomes (CM-Exo) and the exosomes from the ESC-MSCs culture medium (Fig. 3a). Under TEM, the isolated exosomes were round lipid bilayers-vesicles and approximately 30–200 nm in diameter (Fig. 3b). The diameters of isolated exosomes ranged from 38 nm to 169 nm and the mean value was 78 nm (Fig. 3c). The concentration of the isolated exosomes is 176.2 μg/mL (see Additional file 4). The western blot results confirmed the identity of these isolated exosomes by showing thick CD63 and CD9 bands, while there was a weak CD9 band and no CD63 band in the supernatant of cultured medium (Fig. 3d).

**Fig. 4:**
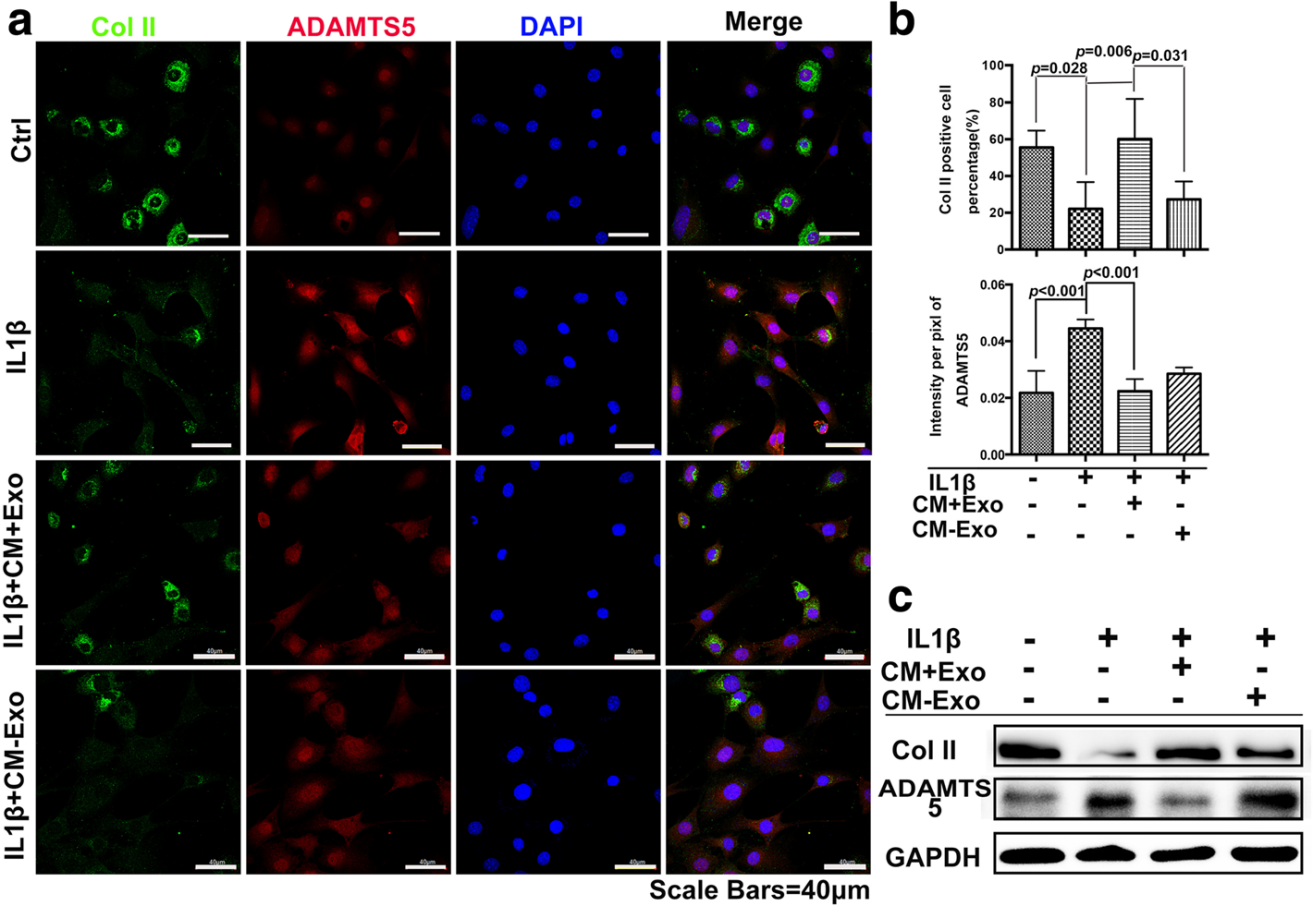
Effects of CM + Exo and CM-Exo on primary chondrocytes phenotype maintenance in the presence of IL-1β. **a** Expression of Col II (*green*) and ADAMTS5 (*red*) by primary chondrocytes treated with CM + Exo and CM-Exo. Scale bar, 40 μm. **b** Percentages of the Col II-positive cells with different treatments and immunofluorescence intensity of ADAMTS5 with different treatments. **c** Expression of Col II and ADAMTS5 by the primary chondrocytes with different treatments. *COL II* collagen type II, *IL-1β*, interleukin 1 beta

Initially, we observed the effects of CM-Exo in maintaining the cartilage matrix. In vitro, immunocytochemistry showed that IL-1β could obviously inhibit the synthesis of Col II and increase the expression of ADAMTS5. However, this effect could be reversed by CM + Exo but not CM-Exo (Fig. 4a). Furthermore, the quantification results showed that the percentage of Col II-positive cells significantly increased from 22.08% ± 13.6% in the IL-1β group to 57.80% ± 7.9% in the CM + Exo group (*p* = 0.0068), which was similar to the control group (55.48% ± 20.7%). By contrast, the percentage of Col II-positive cells in the CM-Exo group was much lower at 27.27% ± 9.2%, which was similar to the IL-1β group (Fig. 4b). The ADAMTS5 immunofluorescence intensity was greatly upregulated after IL-1β treatment (0.044 ± 0.003) compared with the control group (0.02 ± 0.007). The CM + Exo treatment can downregulate the ADAMTS5 immunofluorescence intensity to 0.022 ± 0.004, while the CM-Exo can only downregulate the intensity to 0.028 ± 0.002 (Fig. 4b). These results were doubly confirmed by Western blot analysis (Fig. 4c). The CM + Exo group displayed an obviously thicker Col II band than the CM-Exo group, which was similar to that of the control group. Meanwhile, the CM + Exo group had an obviously lighter ADAMTS5 band than the CM-Exo group, which was similar to that of the control group.

### Effects of exosomes derived from ESC-MSCs on cartilage matrix maintenance

**Fig. 5:**
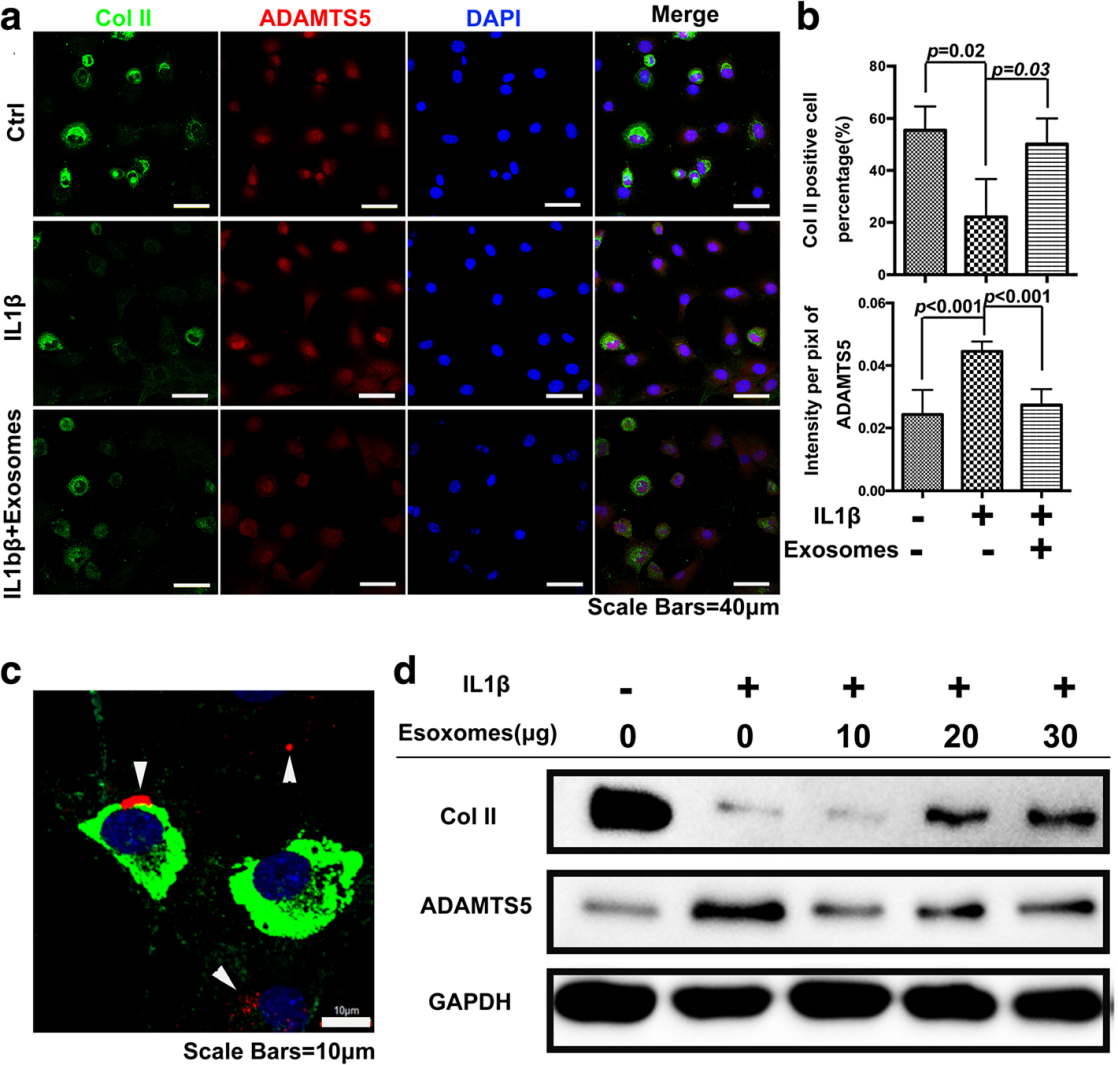
Direct effects of exosomes on primary chondrocyte phenotype maintenance in the presence of IL-1β. **a** Expression of Col II (*green*) and ADAMTS5 (*red*) by primary chondrocytes treated with exosomes. Scale bar, 40 μm. **b** Percentages of the Col II-positive cells with/without the addition of exosomes and immunofluorescence intensity of ADAMTS5 with/without the addition of exosomes. **c** Colocalization of exosomes (*red*), Col II (*green*) and chondrocytes (*blue nuclei*). Scale bars, 10 μm. **d** Expression of Col II and ADAMTS5 by the primary chondrocytes upon addition of different concentrations of exosomes. *COL II* collagen type II, *IL-1β*, interleukin 1 beta

To further confirm the effects of exosomes on cartilage matrix maintenance, the isolated exosomes were directly added into the chondrocytes with IL-1β treatment. Immunocytochemistry showed that the Col II expression in the exosomes group obviously increased, while the ADAMTS5 expression in this group obviously decreased (Fig. 5a). The quantification results confirmed that there were 50.18% ± 8.8% of Col II-positive cells in the exosomes group, which was significantly higher than that in the IL-1β group (22.08% ± 13.6%) (*p* = 0.0323) (Fig. 5b). The immunofluorescence intensity of ADAMTS5 was upregulated from 0.024 ± 0.007 to 0.044 ± 0.003 after IL-1β treatment; but upon addition of exosomes, the intensity was downregulated to 0.027 ± 0.004 (Fig. 5b). Meanwhile, gene expression showed the similar effects of exosome treatment (see Additional file 5). Meanwhile, the DiI-labelled exosomes can be detected within the Col II-expressing chondrocytes (Fig. 5c), thus indicating the colocalization between exosomes and chondrocytes. The western blot results also showed gradually denser Col II bands and gradually fainter ADAMTS5 bands of chondrocytes with increasing dosages of exosomes. (Fig. 5d).

### Alleviation of osteoarthritis by exosomes from ESC-MSCs

**Fig. 6:**
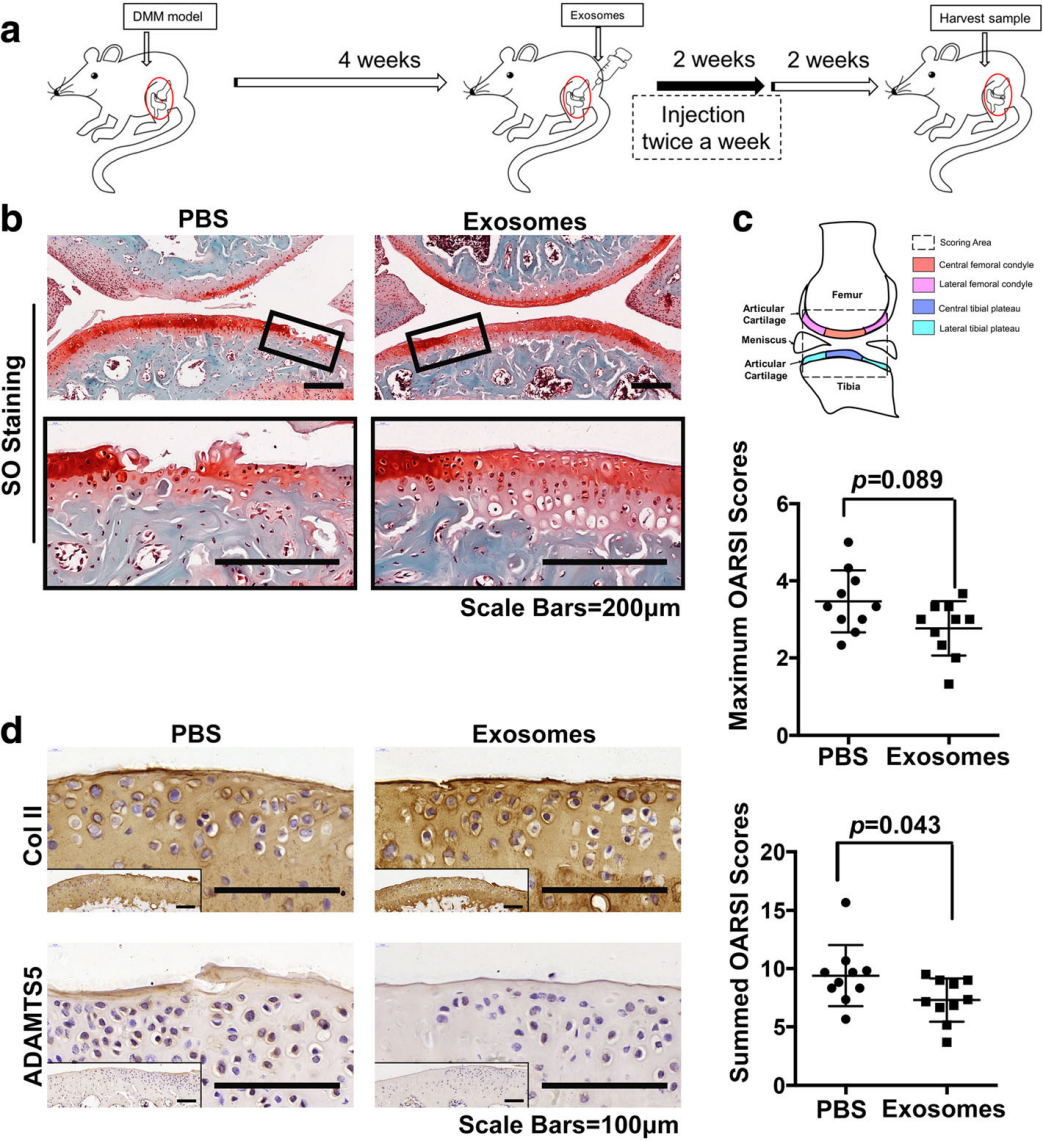
Recovery of cartilage destruction by intra-articular injection of exosomes from ESC-MSCs in a DMM model. **a** Flowchart of the in vivo experiment. **b** Safranin O staining of articular cartilage. *Boxed regions* were magnified and shown in the next line. Scale bars, 200 μm. **c** OARSI scores of cartilage destruction. n = 10 in each group. **d** Immunohistochemical staining of COL II and ADAMTS5. *Boxed regions* show the gross view. Scale bars, 100 μm. *COL II* collagen type II, *OARSI* Osteoarthritis Research Society International

Finally, the effects of ESC-MSC-derived exosomes in alleviating osteoarthritis was evaluated in the DMM mouse model. The injection was started 4 weeks after DMM surgery. Five microliters exosomes or 5 μL PBS were injected into bilateral knee joints of mice in exosomes group and PBS group respectively, every 3 days for 4 weeks (Fig. 6a). The SO staining results showed that the fibrillations had extended to some degree to the calcified cartilage in the PBS group. The maximal OARSI score was 3.5 ± 0.76, and the summed OARSI score was 9.4 ± 2.49. By contrast, the exosomes group exhibited milder OA pathology such as roughened articular surface fibrillations below the superficial layer and some loss of lamina. The maximal OARSI score was 2.7 ± 0.66, and the summed OARSI score was 7.3 ± 1.75 (Fig. 6b) (see Additional files 2 and 6). Both the maximal and total OARSI scores were significantly lower in the exosomes group than the control (Fig. 6c). The immunohistochemistry results showed that the cartilage of the exosomes group displayed much stronger Col II-specific staining, much weaker ADAMTS5-specific staining and aggrecan neoepitope-specific staining than the control group (Fig. 6d) (please refer to Additional file 2).

## Discussion

In this study, we successfully established MSCs from the H1 human ES cell line for OA treatment. These ESC-MSCs fulfilled the minimal criteria of MSCs as defined by the International Society for Cellular Therapy [33], which encompass the plastic adherence property, fibroblastic morphology, molecular profile phenotype, proliferative capacity (see Additional file 4A), and trilineage differentiation potential of these cells. Our results showed that intra-articular injection of ESC-MSCs significantly impeded cartilage destruction in a DMM OA mice model. According to the immunohistochemistry results, the ESC-MSCs can increase Col II (the major cartilage matrix component) expression and decrease ADAMTS5 (aggrecan-degrading enzyme 5) expression in the cartilage matrix.

It is well documented that the secretome of MSCs mediates many of the beneficial therapeutic effects of MSCs on various diseases, and includes a diverse array of cytokines, exosomes, and nuclei acids. Among these, cytokines are the most well studied, but accumulating scientific evidence suggests the important roles of other components. In the past few years, the exosomes derived from MSCs have been reported to have a therapeutic role in various diseases [34, 35]. Hence, we investigated whether the exosomes from ESC-MSCs can alleviate the OA process. To distinguish the activity of exosomes and other soluble components that maintain the chondrocyte phenotype in an inflammatory environment, we harvested the CM + Exo (containing both exosomes and other soluble components), CM-Exo (excluding exosomes) and exosomes by serial centrifugation (Fig. 3a). The following results showed that the CM + Exo decreased ADAMTS5 expression and maintained Collagen type II synthesis by primary chondrocytes under IL-1β treatment in vitro. These effects were almost identical to that of exosomes that play the same role in a dose-dependent pattern. However, the CM-Exo group exhibited lower capacity in maintaining the chondrocyte phenotype. This thus indicates that the exosomes may be one of the major effectors of OA treatment mediated by ESC-MSCs. Additionally, upon tracking the location of exosomes, the exosomes can be found to be colocalized with Col II-expressing chondrocytes, suggesting that these may fuse with chondrocytes and exert a direct effect on these cells.

To confirm the regenerative effects of exosomes in vivo, the exosomes were directly injected into the knee joints of the DMM-induced OA model. The results also confirmed that the exosomes derived from ESC-MSCs effectively prevented the progress of cartilage destruction in an OA model.

Previous studies have shown that the MSCs can be a candidate for osteoarthritis therapy [7, 11, 36]. Most research focus on adult MSCs, which may vary between donors’ physical conditions. Here we attempted to establish MSCs from a promising and consistent new source (ESCs). And these ESC-MSCs displayed similar characteristics to adult MSCs with therapeutic potential in OA. Recent studies have shown that paracrine mechanisms, particularly exosomes are responsible for the therapeutic effects of MSCs mediated OA treatment [37–39]. Tao et al. [38] demonstrated that exosomes derived from human synovial MSCs can promote chondrocyte proliferation and migration. However, only when the exosomes were derived from miR-140-5p overexpressing MSCs, could these exert a regenerative effect in OA treatment. Zhu et al. [39] compared exosomes secreted by induced pluripotent stem cell-derived MSCs and synovial membrane-derived MSCs for OA treatment in a collagenase-induced mouse model. Both studies demonstrated that the exosomes from MSCs could somehow prevent OA progression. And it appears that the exosomes from induced pluripotent stem cell-derived MSCs may be more efficient than the exosomes from synovial membrane-derived MSCs for OA treatment. In this study, we utilized ESC, which is also a pluripotent cell line, as the source of MSCs. Consistent with the published results, the exosomes derived from ESC-MSCs effectively maintained chondrocyte phenotype both in vitro and in vivo. Previously, Zhang et al. reported that the exosomes from ESC-MSCs can enhance cartilage repair in a cartilage defect model [22]. However, there is no direct evidence that has demonstrated the regenerative effects of the exosomes from these cells on OA. In this experiment, we used a DMM mouse model, which is currently the preferred animal OA model resembling the progressive OA pathology. Different from a simple cartilage defect, OA is considered a multifactorial cartilage lesion disease within a chronic inflammatory microenvironment. In our model, the cartilage lesion was caused by accumulated damage on the central weight-bearing area of the medial femoral condyle and the medial tibial plateau. This model is also sufficiently sensitive to allow investigation of new therapeutic strategies that confer significant protection against OA progression. In this model, exosomes by themselves were demonstrated to be able to prevent lesion progression and exert a similar regenerative effect as ESC-MSCs. Further in vitro analysis revealed that this effect was mediated by direct contact between exosomes and chondrocytes and involves balancing the synthesis of extracellular matrix protein Collagen type II with expression of the matrix degradation enzyme ADAMTS5.

Although this study showed that ESC-MSC-derived exosomes play a significant role in preventing OA progression similar to ESC-MSCs, there are still some limitations of our results. We set the two animal experiments sequentially not simultaneously. Meanwhile, the frequency of injection was also different between the ESC-MSC and exosomes groups. Hence, we cannot compare the therapeutic efficacy of exosomes with ESC-MSCs directly, as we cannot exclude other factors that may work together with exosomes to facilitate ESC-MSC-mediated OA treatment. In addition, we only set a single time point and a single dosage in the animal experiment. It would be more clinically relevant to add more time points of intervention in our experiments to explore whether there is a therapeutic window in OA disease. Furthermore, the effective dosage of exosomes needs to be optimized. To date, the active ingredients in exosomes are unknown. As the exosome is a complex carrier which contains lipids, DNA, mRNA, miRNA, LncRNA, and even various proteins, they exhibit therapeutic function through immunomodulation, bioenergetics or biochemical effects in different disease models [19, 21, 24, 34]. Hence, the underlying mechanisms of their therapeutic effects on osteoarthritis need to be further investigated in more detail.

In conclusion, the exosomes are one of the effectors produced by ESC-MSCs to alleviate OA by balancing the synthesis and degradation of cartilage matrix. The study of exosomes in OA therapy has just begun, and our results suggest that exosomes from ESC-MSCs have great potential for OA treatment, which merits further investigation.

## Conclusions

In this study, we confirmed that ESC-MSCs can alleviate OA in a DMM mouse model. The following in vitro study shows that the effects of ESC-MSCs may be dependent on the exosomes in the CM + Exo of ESC-MSCs. We also provided evidence that exosomes can modulate chondrocytes to maintain Col II expression and decrease ADAMTS5 expression under IL-1β treatment. The in vivo study also confirmed the function of exosomes on osteoarthritis treatment in an inflammatory microenvironment. In summary, the exosomes from ESC-MSCs exert a beneficial therapeutic effect on OA by balancing the synthesis and degradation of chondrocyte extracellular matrix (ECM), which in turn provides a new target for OA drug and drug-delivery system development.

## Additional files

Additional file 1: The primers sequence for real-time PCR. (XLSX 46 kb)

Additional file 2: The immunohistochemistry of the joint sample. (A) The SO staining of joints in sham group, scale bars = 200 μm. (B) The IHC staining of aggrecan neoepitope, scale bars = 100 μm. (C) The histology staining of adjacent section, scale bars = 100 μm. (D) The overview of the IHC staining of Col II. (The *solid black arrow* indicate the Col II loss, the *hollow blue arrow* indicates the same background), scale bars = 200 μm. (PNG 1202 kb)

Additional file 3: The OARSI score table of PBS and ESC-MSCs injection group (XLSX 44 kb)

Additional file 4: The detection of ESC-MSCs and exosomes. (A) The proliferation curve of ESC-MSCs tested by CCK8. (B) The analysis of pluripotent marker on ESC and ESC-MSCs. (C) The protein concentration assay of the isolated exosomes. (The *blue line* is the standard curve of protein assay; the *black point* is the detected OD value of exosomes sample). (PNG 116 kb)

Additional file 5: The gene expression related to osteoarthritis upon IL-1β treatment with/without exosomes. (A) The proteases associated with osteoarthritis gene expression related to GAPDH. (B) The Col2a gene expression related to GAPDH. (PNG 367 kb)

Additional file 6: The OARSI score table of PBS and exosomes injection group. (XLSX 40 kb)

## Abbreviations

CM + Exo: Conditioned medium containing exosomes
CM-Exo: Conditioned medium without exosomes
Col II: Collagen type II
DMM: Destabilization of the medial meniscus
ESC-MSCs: Embryonic stem cell-derived mesenchymal stem cells
IL-1β: interleukin 1 beta
MSCs: Mesenchymal stem cells
OA: Osteoarthritis
OARSI: Osteoarthritis Research Society International
qRT-PCR: Quantitative real-time polymerase chain reaction
