# Supplementary figures and images for: Exosomes from embryonic mesenchymal stem cells alleviate osteoarthritis through balancing synthesis and degradation of cartilage extracellular matrix
Source: Stem Cell Res Ther. 2017 Aug 14;8:189. doi: 10.1186/s13287-017-0632-0 (PMC5556343; doi:10.1186/s13287-017-0632-0)

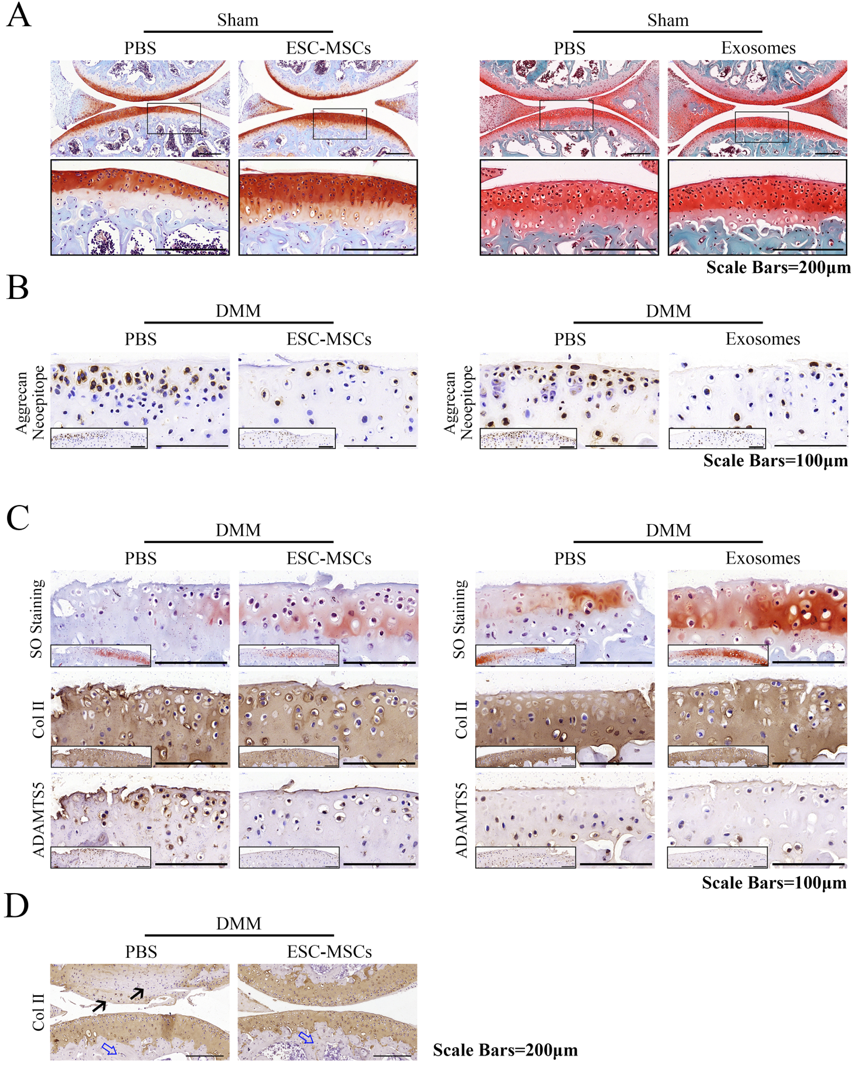

Supplement: Supplementary file 2 — The immunohistochemistry of the joint sample. (A) The SO staining of joints in sham group, scale bars = 200 μm. (B) The IHC staining of aggrecan neoepitope, scale bars = 100 μm. (C) The histology staining of adjacent section, scale bars = 100 μm. (D) The overview of the IHC staining of Col II. (The solid black arrow indicate the Col II loss, the hollow blue arrow indicates the same background), scale bars = 200 μm. (PNG 1202 kb) [file 13287_2017_632_MOESM2_ESM.png]

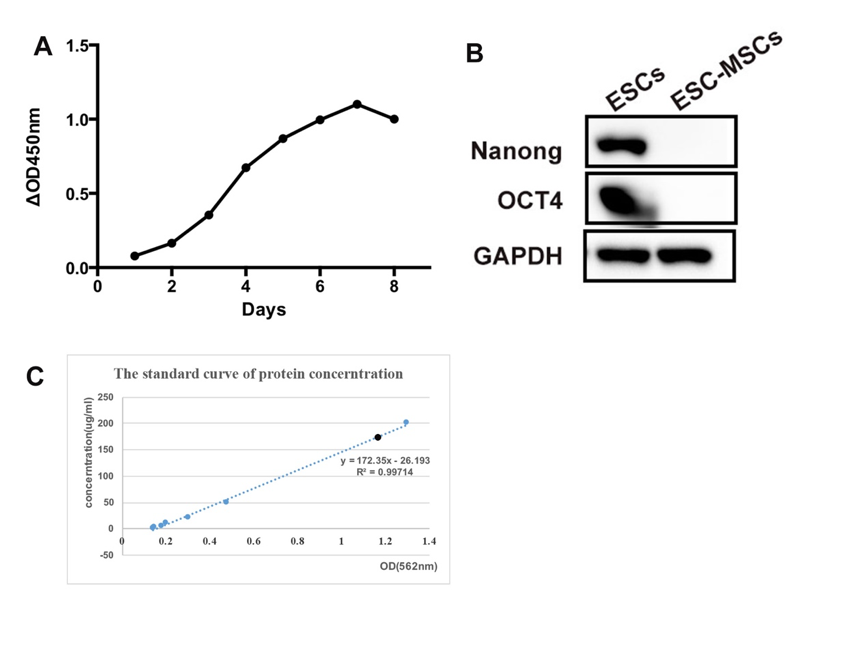

Supplement: Supplementary file 4 — The detection of ESC-MSCs and exosomes. (A) The proliferation curve of ESC-MSCs tested by CCK8. (B) The analysis of pluripotent marker on ESC and ESC-MSCs. (C) The protein concentration assay of the isolated exosomes. (The blue line is the standard curve of protein assay; the black point is the detected OD value of exosomes sample). (PNG 116 kb) [file 13287_2017_632_MOESM4_ESM.png]

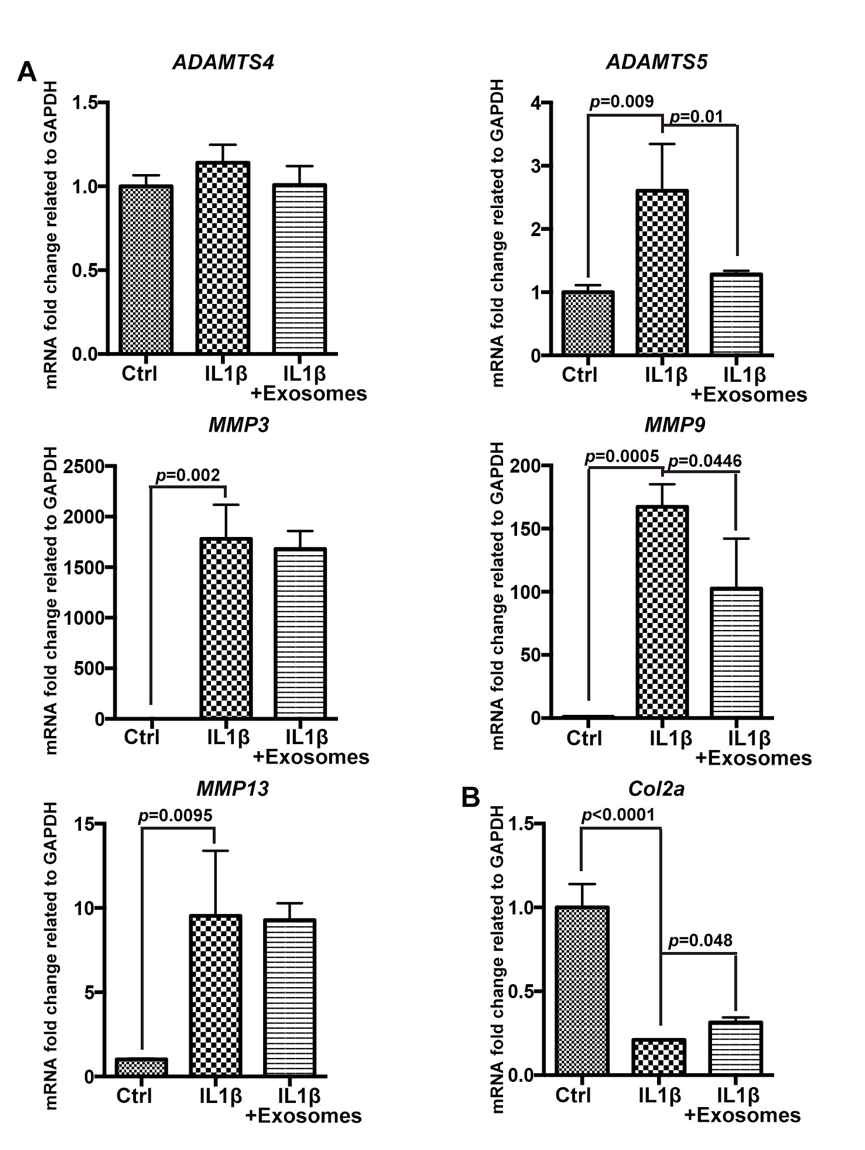

Supplement: Supplementary file 5 — The gene expression related to osteoarthritis upon IL-1β treatment with/without exosomes. (A) The proteases associated with osteoarthritis gene expression related to GAPDH. (B) The Col2a gene expression related to GAPDH. (PNG 367 kb) [file 13287_2017_632_MOESM5_ESM.png]
